# The Metabolic Responses to Aerial Diffusion of Essential Oils

**DOI:** 10.1371/journal.pone.0044830

**Published:** 2012-09-12

**Authors:** Yani Wu, Yinan Zhang, Guoxiang Xie, Aihua Zhao, Xiaolan Pan, Tianlu Chen, Yixue Hu, Yumin Liu, Yu Cheng, Yi Chi, Lei Yao, Wei Jia

**Affiliations:** 1 School of Pharmacy, Shanghai Jiao Tong University, Shanghai, China; 2 School of Agriculture and Biology, Shanghai Jiao Tong University, Shanghai, China; 3 X-omics Research Center for Metabolic Diseases, Shanghai Jiao Tong University Affiliated Sixth People’s Hospital, Shanghai, China; 4 Center for Translational Biomedical Research, University of North Carolina at Greensboro, Kannapolis, North Carolina, United States of America; 5 Center for Instrumental Analysis, Shanghai Jiao Tong University, Shanghai, China; Pennington Biomedical Research Center, United States of America

## Abstract

Anxiety disorders are the most prevalent psychiatric disorders and affect a great number of people worldwide. Essential oils, take effects through inhalation or topical application, are believed to enhance physical, emotional, and spiritual well-being. Although clinical studies suggest that the use of essential oils may have therapeutic potential, evidence for the efficacy of essential oils in treating medical conditions remains poor, with a particular lack of studies employing rigorous analytical methods that capture its identifiable impact on human biology. Here, we report a comprehensive gas chromatography time-of-flight mass spectrometry (GC-TOFMS) based metabonomics study that reveals the aromas-induced metabolic changes and the anxiolytic effect of aromas in elevated plus maze (EPM) induced anxiety model rats. The significant alteration of metabolites in the EPM group was attenuated by aromas treatment, concurrent with the behavioral improvement with significantly increased open arms time and open arms entries. Brain tissue and urinary metabonomic analysis identified a number of altered metabolites in response to aromas intervention. These metabolic changes included the increased carbohydrates and lowered levels of neurotransmitters (tryptophan, serine, glycine, aspartate, tyrosine, cysteine, phenylalanine, hypotaurine, histidine, and asparagine), amino acids, and fatty acids in the brain. Elevated aspartate, carbohydrates (sucrose, maltose, fructose, and glucose), nucleosides and organic acids such as lactate and pyruvate were also observed in the urine. The EPM induced metabolic differences observed in urine or brain tissue was significantly reduced after 10 days of aroma inhalation, as noted with the loss of statistical significance on many of the metabolites in the aroma-EPM group. This study demonstrates, for the first time, that the metabonomics approach can capture the subtle metabolic changes resulting from exposure to essential oils and provide the basis for pinpointing affected pathways in anxiety-related behavior, which will lead to an improved mechanistic understanding of anxiolytic effect of essential oils.

## Introduction

Anxiety is a substantive public health problem and affects a great number of people worldwide [1]. An increasing number of studies have shown that essential oils can decrease anxiety-related behavior in humans and animals [2], [3] and demonstrated that the aromatic plant-derived essential oils exhibit a variety of biological properties, such as mood enhancement, pain relief, and improved cognitive function, which is being used in complementary and traditional medicine units as well as primary care settings [4]. The inhaled aromas from the essential oils are believed to be useful for a vast array of symptoms and conditions and a large body of published literature has generally focused on the human brain and emotions [5], [6]. Several studies suggest that neurotransmitters 5-hydroxytryptamine and dopamine may be modulated with the anti-anxiety effect by essential oils from rose, lavender, lemon and peppermint [7], [8]. Other studies investigated the effects of various plant-derived or synthetic odors on task performance, reaction time, and autonomic parameters or evaluated the direct effects of odors on the brain via electroencephalogram patterns and functional imaging studies [6]. These studies have consistently shown that odors can produce specific effects on human neuropsychological and autonomic function, suggesting that essential oils has beneficial effects in the context of stressful and adverse psychological conditions.

Despite the numerous results demonstrating beneficial effects on mood and relaxation observed in behavioral and emotional studies, evidence for the efficacy and mechanistic understanding of essential oils in treating medical conditions remains poor, with a particular lack of studies employing rigorous methodology [9], [10]. Some benefits that have been linked to essential oils intervention, such as relaxation and clarity of mind, may arise from the placebo effect rather than from any actual physiological effect.

Here, we report a metabonomic study designed to evaluate the effect of an essential oil preparation in Wistar rats. Brain tissues and urine samples were collected and analyzed by gas chromatography time-of-flight mass spectrometry (GC-TOFMS) to obtain significant endogenous metabolite markers of aromas-exposure. We also evaluated the anxiolytic effect of aromas in EPM model by comparing rats exposed to essential oil against rats without any intervention.

## Materials and Methods

### Chemicals and Materials

The essential oil used in the study is a classical formula [11], prepared from 4 aromatic plants, *Lavandula angustifolia, Salvia sclarea L., Santalum album,* and *Citrus sinensis* following the published procedure [12]. The constituents of the essential oil were assayed by gas chromatography (GC) and GC-mass spectrometry (GC-MS) and listed in **Table S1**. Acetonitrile and methanol of HPLC grade were obtained from Merck Chemicals (Darmstadt, Germany). Analytical-grade methanol was obtained from the Shanghai Lin Feng Chemical Reagent Co, Ltd. (China). All aqueous solutions were prepared with ultrapure water produced by a Milli-Q system (18.2 MΩ, Milipore, Bedford, MA). Chloroform was analytical grade and purchased from China National Pharmaceutical Group Corporation (Shanghai, China). L-2-chlorophenylalanine was purchased from Intechem Tech. Co. Ltd. (Shanghai, China). BSTFA (1% TMCS), heptadecanoic acid, and methoxyamine were purchased from Sigma-Aldrich (St. Louis, MO).

### Animal Treatment and Sampling

The animal study was conducted in accordance with Chinese national legislation and local guidelines and performed at the Centre of Laboratory Animals, Shanghai Jiao Tong University, China. All animal protocols were reviewed and approved by the ethics committee of the School of Pharmacy, Shanghai Jiao Tong University, China.

A total of 40 female Wistar rats of body weight 160–180 g were obtained from Shanghai Laboratory Animal Co. Ltd. (SLAC, China). Each rat was housed in one metabolic cage and kept in a barrier system with regulated temperature (20±2°C) and humidity (60±10%), on a 12/12 h light/dark cycle. All experimental rats received rat chow and water *ad libitum* throughout the experiment, and the average weight gain was monitored. After a one week acclimatization, the 40 rats were randomly divided into four groups: control group (n = 10), without smelling essential oil and no elevated plus maze (EPM) test during the experiment; aroma group (n = 10), inhaled essential oil for 45 min every day for 10 days; aroma-EPM group (n = 10), inhaled essential oil for 45 min every day for 10 days and underwent EPM test on day eleven; EPM group (n = 10), underwent EPM test on day eleven without smelling essential oil. At the end of 11^th^ day, urine samples were collected and stored at −80°C pending GC-MS analysis. All the rats were anesthetized and the brain tissues were collected and weighed.

### Elevated Plus-maze

The elevated plus maze, a well-established animal model of anxiety, was performed on a maze composed of two opposing transparent open (50×10 cm) and two opposing closed arms (50×10×40 cm), and elevated 0.5 m above the floor. Once placed on the center of the maze (facing a closed arm), rats were observed for 5 min. Open and closed arm entries were noted when a rat had all four paws in a given arm. The primary index for increased anxiety-like response in this test is a decrease in the proportion of time spent in the open arms (open time/open + closed time) and/or the proportion of entries made onto the open arms of the maze (open entries/open + closed entries) [13].

### Urine Sample Preparation for GC-TOFMS Analysis

Urine metabolites were chemically derivatized prior to mass spectrometry analysis following our previously published procedure with minor modifications [14]. An aliquot of 100 µL urine sample was spiked with two internal standard solutions (10 µL of L-2-chlorophenylalanine in water, 0.3 mg/mL; 10 µL of heptadecanoic acid in methanol, 1 mg/mL) and vortexed for 10 s. The mixed solution was extracted with 300 µL of methanol/chloroform (3∶1) and vortexed for 30 s. After storing for 10 min at −20°C, the samples were centrifuged at 12 000 g for 10 min. An aliquot of the 300-µL supernatant was transferred to a glass sampling vial to vacuum-dry at room temperature. The residue was derivatized using a two-step procedure. First, 80 µL of methoxyamine (15 mg/mL in pyridine) was added to the vial and kept at 30°C for 90 min, followed by 80 µL of BSTFA (1% TMCS) at 70°C for 60 min.

### Brain Tissues Sample Preparation for GC-TOFMS Analysis

Tissue samples were prepared according to our previous published method with minor modification [15]. Briefly, each tissue sample (50 mg) was extracted with 250 µL mixed solvent (chloroform: methanol: water = 1∶2.5∶1, v/v/v) and homogenized for 2 min. After storing for 20 min at −20°C, the samples were centrifuged at 12, 000 g for 10 min. A total of 150 µL of aqueous supernatant was transferred to a GC vial containing two internal standards, L-2-chlorophenylalanine (10 µL, 0.3 mg/mL) and heptadecanoic acid (10 µL, 1.0 mg/mL). The deposit was re-homogenized with a T10 basic homogenizer (IKA, Staufen, Germany) for 30 sec at 0°C after adding 250 µL of methanol. Then the samples were centrifuged at 12 000 g for 10 min. After a second centrifugation, another 150 µL aliquot of supernatant was added to the mixture in the GC vial and vacuum dried. The residue was derivatized using a two-step procedure: first, 80 µL of methoxyamine (15 mg/mL in pyridine) was added to the vial and kept at 30°C for 90 min, and then followed by addition of 80 µL of BSTFA (1% TMCS) to the vial and maintained at 70°C for 60 min.

### GC-TOFMS Analysis

A 1 µL aliquot of the derivatized solution was injected in splitless mode into an Agilent 6890N gas chromatograph coupled with a Pegasus HT time-of-flight mass spectrometer (Leco Corporation, St. Joseph, MI). Separation was achieved on a DB-5 ms capillary column (30 m×250 µm i.d., 0.25 µm film thickness; (5%-phenyl)-methylpolysiloxane bonded and cross-linked; Agilent J&W Scientific, Folsom, CA), with helium as the carrier gas at a constant flow rate of 1.0 mL/min. The temperature of injection, transfer interface, and ion source was set to 270, 260, and 200°C, respectively. The GC temperature programming was set to 2 min isothermal heating at 80°C, followed by 10°C/min oven temperature ramps to 180°C, 5°C/min to 240°C, and 25°C/min to 290°C, and a final 9 min maintenance at 290°C. Electron impact ionization (70 eV) at full scan mode (m/z 30–600) was used, with an acquisition rate of 20 spectra/s in the TOFMS setting.

### GC-TOFMS Data Analysis

The acquired MS files from GC-TOFMS analysis were exported in NetCDF format by ChromaTOF software (v3.30, Leco Co., CA). CDF files were extracted using custom scripts (revised Matlab toolbox hierarchical multivariate curve resolution (H-MCR), developed by Par Jonsson et al. [16], [17] in the MATLAB 7.0 (The MathWorks,Inc.) for data pretreatment procedures such as baseline correction, denoising, smoothing, alignment, time-window splitting, and multivariate curve resolution (based on multivariate curve resolution algorithm) [17]. The resulting three-dimensional data set included sample information, peak retention time and peak intensities. Internal standards and any known artificial peaks, such as peaks caused by noise, column bleed and BSTFA derivatization procedure, were removed from the data set. The resulting data was mean centered and unit variance scaled during chemometric data analysis in the SIMCA-P+12.0 Software package (Umetrics, Umeå, Sweden). Orthogonal partial least squares-discriminant analysis (OPLS-DA) was carried out to discriminate between different groups. On the basis of a variable importance in the projection (VIP) threshold of 1 from the OPLS-DA model, a number of metabolites responsible for the difference in the metabolic profiles between two groups could be obtained. In parallel, the metabolites identified by the OPLS-DA model were validated at a univariate level using the Student’s t test with the critical p-value set to 0.05. The resultant p values for all metabolites were subsequently adjusted to account for multiple testing. The false discovery rate (FDR) method of Pike [18] was used to perform the adjustment. The corresponding fold change shows how these selected differential metabolites varied between groups. Additionally, compound identification was performed by comparing the mass fragments with NIST 05 Standard mass spectral databases in NIST MS search 2.0 (NIST, Gaithersburg, MD) software with a similarity of more than 70% and finally verified by available reference compounds.

## Results

### Body Weight

To determine whether there were treatment effects on general growth, rats were weighed on the 1^st^ day and 10^th^ day: just after the initiation of and immediately after the end of the 10-day aromas exposure. The body weight of the rats increased steadily during the experiment. There was no significant difference between the control group and aroma group on day one (p>0.8), while significant effects of aromas exposure on body weight (*p*<0.02) were found that rats weigh more after 10 days exposure to essential oil (data not shown).

### EPM Test

**Figure 1:**
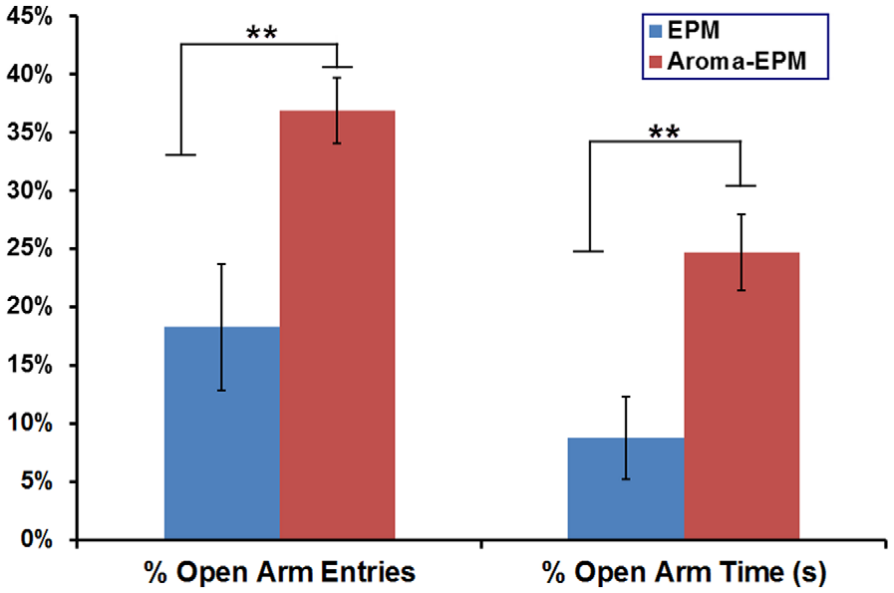
Open arm activity in the elevated plus maze (EPM) displayed by female Wistar rats with and without aromas exposure. Data are expressed as mean (±SEM) of the percentage of open arm entries made and percentage of time spent in the open arm by rats (n = 10). Asterisks denote a significant main effect of treatment, **P<0.01.

The EPM group and aroma-EPM group underwent the EPM test on the 11^th^ day to evaluate their behavior. The percent of open arm entries (%OE) and percent open arm time (%OT) are shown in **Figure 1**. Inhalation of essential oil significantly increased the open arms time and open arms entries (p<0.01).

### Metabolic Profile of GC-TOFMS Analysis

**Figure 2:**
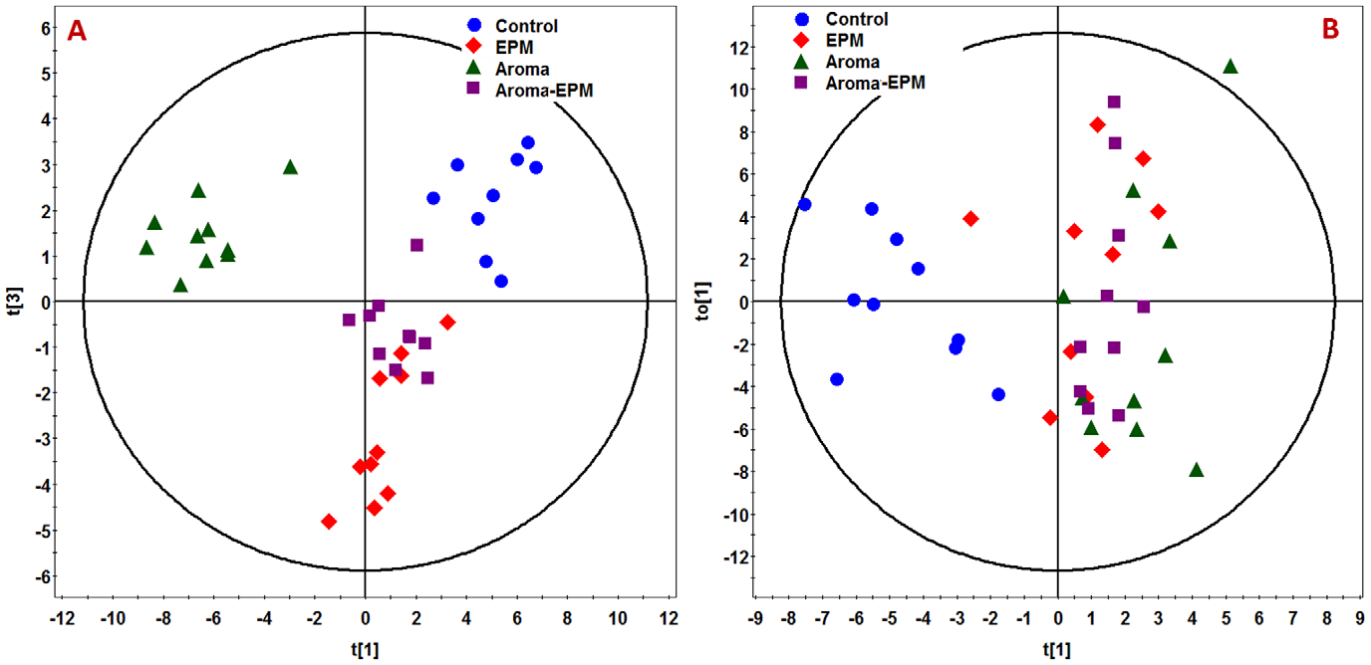
Metabolic profiles depicted by OPLS-DA scores plot of GC-TOFMS spectral data from the (A) brain tissue and (B) urine of control group, EPM group, aroma group, and aroma-EPM group.

A wide range of neurotransmitters, carbohydrates, amino acids, organic acids, fatty acids, nucleotides, and alcohols were detected using GC-TOF MS analysis of brain tissues and urine. Among a total of 314 and 245 chromatographic features obtained from the GC-TOFMS spectra of urine and brain tissue samples, 133 and 108 metabolites were identified with NIST 05 standard mass spectral databases with a similarity >70% and 83 and 75 were further verified by available reference standards respectively. **Figure 2** illustrates the scores plots of OPLS-DA model of the subjects from control group, EPM group, aroma group, aroma-EPM group. EPM group, aroma group, aroma-EPM group were all clearly separated from the controls.

**Figure 3:**
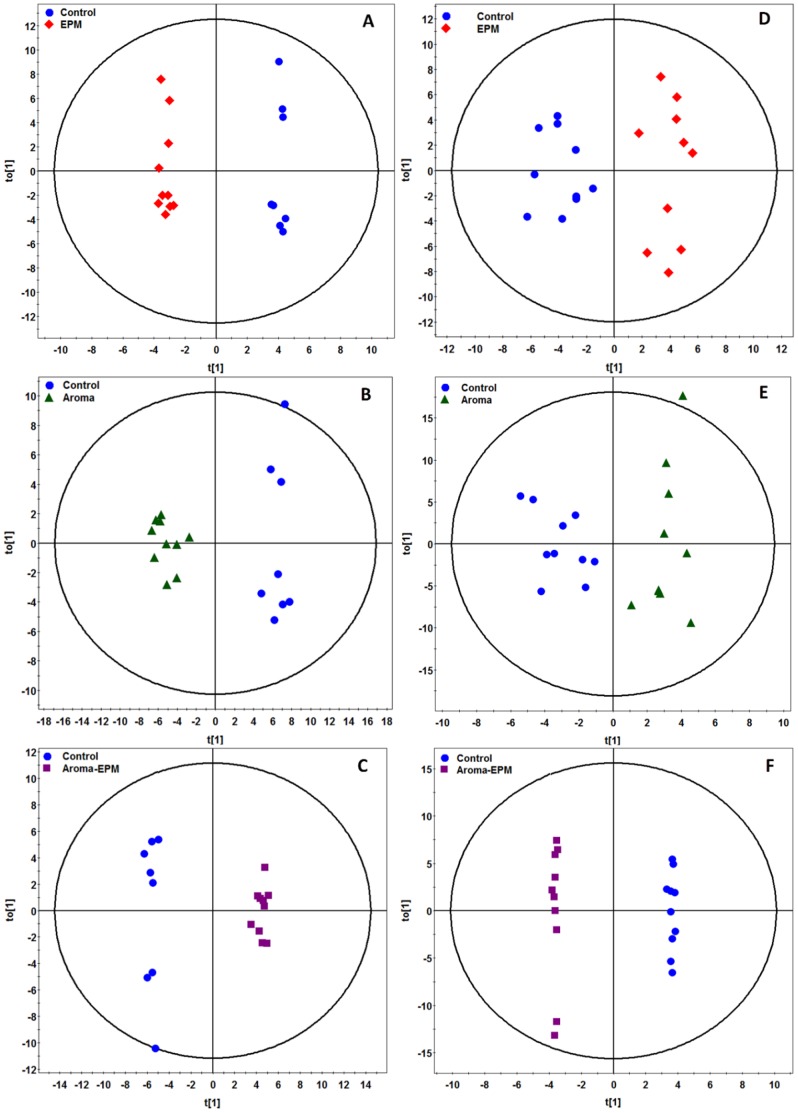
Metabolic profiles depicted by OPLS-DA scores plot of GC-TOFMS spectral data from the (A–C) brain tissue and (D–F) urine of control group, EPM group, aroma group, and aroma-EPM group.

We selected the differentially expressed brain tissue and urine metabolites in the aroma group, EPM group, and aroma-EPM group relative to control group based on the VIP values (VIP>1) by OPLS-DA models (urine: R2X = 0.633, R2Y = 0.877, Q2(cum) = 0.436; R2X = 0.638, R2Y = 0.902, Q2(cum) = 0.371; R2X = 0.803, R2Y = 0.999, Q2(cum) = 0.832; brain: R2X = 0.702, R2Y = 0.994, Q2(cum) = 0.921; R2X = 0.804, R2Y = 0.990, Q2(cum) = 0.686; R2X = 0.723, R2Y = 0.995, Q2(cum) = 0.964), respectively (**Figures 3A–F**) constructed with the identified metabolites. Univariate statistical analysis, Student’s t test, was performed on metabolites identified from GC-TOFMS analysis of tissues and urine samples to evaluate their significance. Differentially expresssed metabolites in brain tissue and urine were obtained with a *p* value less than 0.05 (**Tables 1** **–** **2**). All these metabolites remained statistically significant after multiple testing.

**Table 1:** List of differential metabolites in brain tissues and urine in aroma group relative to control group.

Sample	Classes	Metabolites	VIP^1^	FC^2^	P^3^	Adjusted p^4^
**Brain**	Alcohols	Arabitol	1.37	0.49	7.65E−04	1.99E−03
		Glyceraldehyde	1.43	0.63	3.81E−04	1.40E−03
		Glycerol	1.19	0.77	5.48E−03	7.73E−03
	Amino acids	Proline	1.45	0.37	2.60E−04	1.21E−03
		Isoleucine	1.34	0.41	1.21E−03	2.78E−03
		Valine	1.28	0.43	2.40E−03	4.25E−03
		Alanine	1.57	0.55	3.32E−05	7.10E−04
		Pantothenate	1.24	0.57	3.55E−03	5.43E−03
		Threonine	1.48	0.58	1.77E−04	1.08E−03
		lysine	1.14	0.58	8.37E−03	1.12E−02
	Carbohydrates	Arabinose	1.30	0.66	1.74E−03	3.55E−03
		N-Acetyl-beta-D-mannosamine	1.01	0.69	2.31E−02	2.56E−02
		N-Acetylglucosamine	1.06	0.88	1.63E−02	2.00E−02
		Threitol	1.05	1.29	1.70E−02	2.04E−02
		N-acetylglutamine	1.44	1.36	2.86E−04	1.21E−03
		Glucose	1.29	1.41	1.97E−03	3.87E−03
		Galactose	1.00	1.49	2.42E−02	2.61E−02
		Mannose	1.29	1.68	2.15E−03	4.06E−03
		Glucose-6-phosphate	1.03	1.71	2.06E−02	2.40E−02
		Glucuronolactone	1.51	1.97	1.03E−04	7.10E−04
		Glucose-5-phosphate	1.27	3.34	2.50E−03	4.29E−03
	Fatty acids	Methyl arachidonate	1.73	0.37	4.11E−07	2.26E−05
		Linolate	1.45	0.47	2.68E−04	1.21E−03
		Docosahexaenoate	1.32	0.55	1.48E−03	3.25E−03
		Oleate	1.25	0.58	3.11E−03	5.18E−03
		Palmitate	1.24	0.60	3.41E−03	5.36E−03
		Stearate	1.28	0.64	2.22E−03	4.06E−03
		Palmitoleate	1.30	0.66	1.74E−03	3.55E−03
		Arachidonate	1.21	0.68	4.61E−03	6.67E−03
	Neurotransmitters	Histidine	1.21	0.28	4.41E−03	6.56E−03
		Phenylalanine	1.38	0.40	6.83E−04	1.98E−03
		Tryptophan	1.53	0.47	7.22E−05	7.10E−04
		Asparagine	1.02	0.47	2.09E−02	2.40E−02
		Tyrosine	1.41	0.50	4.75E−04	1.54E−03
		Serine	1.52	0.55	7.90E−05	7.10E−04
		Hypotaurine	1.37	0.67	8.34E−04	1.99E−03
		Glycine	1.47	0.71	1.98E−04	1.09E−03
		Aspartate	1.44	0.73	3.27E−04	1.28E−03
		Cysteine	1.39	0.78	6.11E−04	1.87E−03
		Histamine	1.42	1.97	4.30E−04	1.48E−03
	Nucleosides	Uracil	1.15	0.62	7.60E−03	1.04E−02
		Xanthine	1.38	0.65	7.24E−04	1.99E−03
		Hypoxanthine	1.51	0.65	9.46E−05	7.10E−04
		Inosine	1.53	2.17	7.28E−05	7.10E−04
	Organic acids	Citrate	1.51	0.41	1.02E−04	7.10E−04
		Citrulline	1.07	0.46	1.53E−02	1.91E−02
		Aminomalonate	1.37	0.61	7.96E−04	1.99E−03
		Thiazolidine-4-carboxylate	1.10	0.62	1.20E−02	1.54E−02
		Pyruvate	1.14	0.69	8.67E−03	1.14E−02
		Malonate	1.25	1.85	3.22E−03	5.22E−03
**Urine**	Amino acids	Methionine	1.92	2.46	1.39E−02	4.48E−02
		Lysine	1.86	1.62	1.84E−02	4.48E−02
	Carbohydrates	Fructose	1.63	1.49	4.24E−02	4.65E−02
		Glucose	1.61	1.51	4.51E−02	4.65E−02
		Maltose	1.67	2.13	3.75E−02	4.56E−02
		Sucrose	1.82	3.11	2.15E−02	4.48E−02
	Fatty acids	Butyl palmitate	1.74	3.98	2.91E−02	4.48E−02
	Neurotransmitters	Aspartate	2.19	3.72	3.87E−03	4.48E−02
	Nucleosides	Adenine	1.76	2.06	2.63E−02	4.48E−02
		Uridine	1.82	9.76	2.13E−02	4.48E−02
	Organic acids	Parabanate	1.69	1.60	3.44E−02	4.50E−02
		Lactate	1.71	1.62	3.16E−02	4.48E−02
		Pyruvate	1.82	1.68	2.09E−02	4.48E−02
		4-Hydroxybenzoate	1.60	2.22	4.65E−02	4.65E−02
		Benzoylformate	1.96	2.64	1.18E−02	4.48E−02
	Others	2,8-Dihydroxyquinoline	1.76	4.34	2.71E−02	4.48E−02
		Phosphate	1.79	0.68	2.43E−02	4.48E−02

Note: ^1^Variable importance in the projection (VIP) was obtained from OPLS-DA model with a threshold of 1.0;

2 fold change (FC) was obtained by comparing those metabolites in aroma group to control group;

3 *p* values were calculated from Student’s t test;

4 Adjusted for multiple testing with FDR control [18]. FC with a value >1 indicates a relatively higher concentration present in aroma group while a value <1 means a relatively lower concentration as compared to the controls.

**Table 2:** List of differential metabolites in brain tissues and urine in EPM group relative to controls, and aroma-EPM group relative to controls.

			EPM vs. Control				Aroma-EPM vs. Control			
Sample	Classes	Metabolites	VIP^1^	FC^2^	P^3^	Adjusted p^4^	VIP^1^	FC^5^	P^3^	Adjusted p^4^
Brain	Alcohols	Arabitol	1.48	0.70	3.39E−02	4.36E−02	1.41	0.57	3.01E−03	1.27E−02
		Glyceraldehyde	1.28	0.79	3.99E−02	4.49E−02	1.40	1.42	3.29E−03	1.27E−02
		Glycerol	1.19	0.85	4.32E−02	4.49E−02	0.65	1.12	2.21E−01	3.13E−01
		Myo-inositol	1.35	0.85	3.00E−02	4.28E−02	0.82	1.13	1.15E−01	1.94E−01
	Amino acids	Isoleucine	1.35	0.64	1.12E−02	2.83E−02	0.62	0.78	2.45E−01	3.31E−01
		Valine	1.29	0.64	1.51E−02	2.91E−02	0.76	0.73	1.44E−01	2.16E−01
		Proline	1.38	0.67	1.33E−02	2.83E−02	1.04	0.67	4.01E−02	7.73E−02
		Cysteine	2.34	0.69	1.03E−05	2.78E−04	1.75	0.66	4.54E−05	4.09E−04
		Threonine	1.48	0.77	5.95E−03	2.83E−02	1.20	0.75	1.54E−02	4.17E−02
		Alanine	1.53	0.78	8.13E−03	2.83E−02	1.17	0.80	1.90E−02	4.66E−02
	Carbohydrates	Ribose-5-phosphate	1.80	0.43	7.77E−03	2.83E−02	1.34	0.41	5.67E−03	1.70E−02
		Galactofuranose	1.32	0.72	4.84E−02	4.84E−02	0.54	0.83	3.12E−01	3.84E−01
		Arabinose	1.67	0.76	2.03E−02	3.66E−02	0.42	0.93	4.29E−01	4.63E−01
		Glucose-6-phosphate	1.02	1.41	4.25E−02	4.49E−02	2.06	7.11	3.39E−09	9.15E−08
		Glucose-5-phosphate	1.03	1.62	2.78E−02	4.28E−02	1.99	18.75	8.12E−08	1.10E−06
	Neurotransmitters	Phenylalanine	1.38	0.65	1.14E−02	2.83E−02	1.05	0.65	3.77E−02	7.73E−02
		Tryptophan	1.29	0.75	8.46E−03	2.83E−02	0.54	0.84	3.13E−01	3.84E−01
		Tyrosine	1.23	0.75	1.36E−02	2.83E−02	0.78	0.77	1.34E−01	2.13E−01
		Serine	1.34	0.79	9.73E−03	2.83E−02	1.14	0.77	2.30E−02	5.18E−02
		Glycine	1.67	0.80	3.09E−03	2.78E−02	0.43	0.94	4.20E−01	4.63E−01
		Aspartate	1.60	0.83	2.72E−03	2.78E−02	0.29	0.95	5.95E−01	6.18E−01
		GABA	1.43	0.92	2.26E−02	3.82E−02	1.47	1.25	1.70E−03	9.18E−03
	Organic acids	Gluconate	1.47	0.74	3.05E−02	4.28E−02	0.49	1.11	3.62E−01	4.25E−01
		Oxalate	1.73	0.80	3.88E−02	4.49E−02	0.10	1.01	8.47E−01	8.47E−01
		Lactate	1.36	0.85	3.17E−02	4.28E−02	1.63	1.35	2.90E−04	1.96E−03
	Others	Phosphorate	1.30	0.77	1.21E−02	2.83E−02	1.36	1.40	4.73E−03	1.60E−02
Urine	Alcohols	Xylitol	1.37	1.85	3.14E−02	4.42E−02	1.19	1.26	9.66E−02	9.82E−02
		L-Threitol	1.31	1.33	4.19E−02	4.71E−02	1.45	1.31	3.89E−02	7.32E−02
		Normetanephrine	1.48	1.45	1.85E−02	4.09E−02	1.70	1.51	1.28E−02	4.11E−02
	Amino acids	Alanine	1.52	1.30	1.52E−02	4.05E−02	1.77	1.30	9.12E−03	4.11E−02
		Methionine	1.40	1.70	2.83E−02	4.42E−02	1.36	1.70	5.49E−02	7.32E−02
		Lysine	1.61	1.53	9.42E−03	4.05E−02	1.19	1.43	9.82E−02	9.82E−02
		Valine	1.28	2.28	4.61E−02	4.76E−02	0.43	1.37	5.66E−01	3.14E−01
	Carbohydrates	Fructose	1.43	1.52	2.36E−02	4.42E−02	1.73	1.56	1.13E−02	4.11E−02
		Glucose	1.60	1.54	9.91E−03	4.05E−02	1.38	1.30	5.00E−02	7.32E−02
		Xylulose	1.42	1.39	2.49E−02	4.42E**−**02	1.38	1.32	5.09E**−**02	7.32E**−**02
		1,5-Anhydroglucitol	1.54	2.48	1.38E**−**02	4.05E**−**02	0.38	1.11	6.11E**−**01	3.15E**−**01
		Maltose	1.50	1.88	1.73E**−**02	4.09E**−**02	0.97	1.47	1.80E**−**01	1.50E**−**01
		Sucrose	1.52	2.19	1.57E**−**02	4.05E**−**02	1.36	2.03	5.41E**−**02	7.32E**−**02
	Fatty acids	Oleate	1.41	1.55	2.67E**−**02	4.42E**−**02	0.75	1.27	3.09E**−**01	2.06E**−**01
	Indoles	1H-Indole	1.54	1.61	1.37E**−**02	4.05E**−**02	1.13	1.25	1.16E**−**01	1.04E**−**01
	Ketone	Hydroxyurea	1.36	2.76	3.29E**−**02	4.43E**−**02	0.64	2.13	3.85E**−**01	2.37E**−**01
	Neurotransmitters	Glutamate	1.60	1.66	1.01E**−**02	4.05E**−**02	1.17	1.40	1.05E**−**01	9.88E**−**02
		Aspartate	1.46	1.97	2.04E**−**02	4.22E**−**02	1.42	2.33	4.31E**−**02	7.32E**−**02
		Phenylalanine	1.38	1.56	3.05E**−**02	4.42E**−**02	0.80	1.29	2.75E**−**01	1.91E**−**01
	Nucleosides	Adenine	1.87	2.17	1.67E**−**03	4.05E**−**02	1.26	1.45	7.83E**−**02	9.64E**−**02
		Thymine	1.30	3.20	4.40E**−**02	4.71E**−**02	0.42	1.65	5.69E**−**01	3.14E**−**01
	Organic acids	Pyruvate	1.34	1.79	3.58E**−**02	4.44E**−**02	1.82	1.79	6.85E**−**03	4.11E**−**02
		4-Hydroxybenzoate	1.68	2.17	6.20E**−**03	4.05E**−**02	1.21	2.30	8.99E−02	9.82E−02
		Saccharate	1.30	1.27	4.41E−02	4.71E−02	0.83	1.20	2.58E−01	1.91E−01
		Gluconate	1.58	1.33	1.10E−02	4.05E−02	0.72	1.15	3.26E−01	2.09E−01
		Aminomalonate	1.35	3.18	3.51E−02	4.44E−02	0.96	1.81	1.88E−01	1.50E−01
		Benzoylformate	1.27	1.96	4.95E−02	4.95E−02	1.57	3.78	2.36E−02	6.30E−02
		m-Hydroxyphenylacetate	1.55	2.70	1.32E−02	4.05E−02	0.81	1.60	2.72E−01	1.91E−01
		Lactate	1.38	1.76	3.04E−02	4.42E−02	0.39	1.09	5.98E−01	3.15E−01
		5-Hydroxyindole-3-acetate	1.30	1.78	4.37E−02	4.71E−02	0.50	1.18	4.96E−01	2.94E−01
	Others	2,8-Dihydroxyquinoline	1.63	4.14	8.55E−03	4.05E−02	1.76	4.39	9.76E−03	4.11E−02

Note: ^1^Variable importance in the projection (VIP) was obtained from OPLS-DA model with a threshold of 1.0;

2 fold change (FC) was obtained by comparing those metabolites in EPM group to control group;

3 *p* values were calculated from Student’s t test;

4 Adjusted for multiple testing with FDR control [18];

5 FC was obtained by comparing those metabolites in aroma-EPM group to control group. FC with a value >1 indicates a relatively higher concentration present in EPM group or aroma-EPM group while a value <1 means a relatively lower concentration as compared to the controls.

### Daily Exposure to Aromas Induces Significant Metabolic Changes

We detected 50 differentially expressed metabolites induced by aromas inhalation in rat brain tissue (**Table 1**), including a number of neurotransmitters, carbohydrates, alcohols, nucleosides, amino acids and fatty acids. As compared to controls, rats exposed to aromas were characterized by higher levels of carbohydrates (threitol, glucose-6-phosphate, glucose, glucuronolactone, N-acetylglutamine, mannose, and glucose-5-phosphate), lower levels of neurotransmitters (tryptophan, serine, glycine, aspartate, tyrosine, cysteine, phenylalanine, hypotaurine, histidine, and asparagine), amino acids (alanine, threonine, proline, pantothenate, and lysine), alcohols (glyceraldehyde, arabitol, and glycerol), and fatty acids (methyl arachidonate, linolate, docosahexaenoate, palmitoleate, stearate, oleate, palmitate, arachidonate).

Analysis of urine metabolic profiles reveals significant metabolic responses induced by aromas and seventeen differentially expressed metabolites including elevated neurotransmitter (aspartate), carbohydrates (sucrose, maltose, fructose, and glucose), nucleosides (adenine and uridine) and organic acids such as lactate and pyruvate.

### Significant Metabolic Changes is Associated with EPM

**Figure 4:**
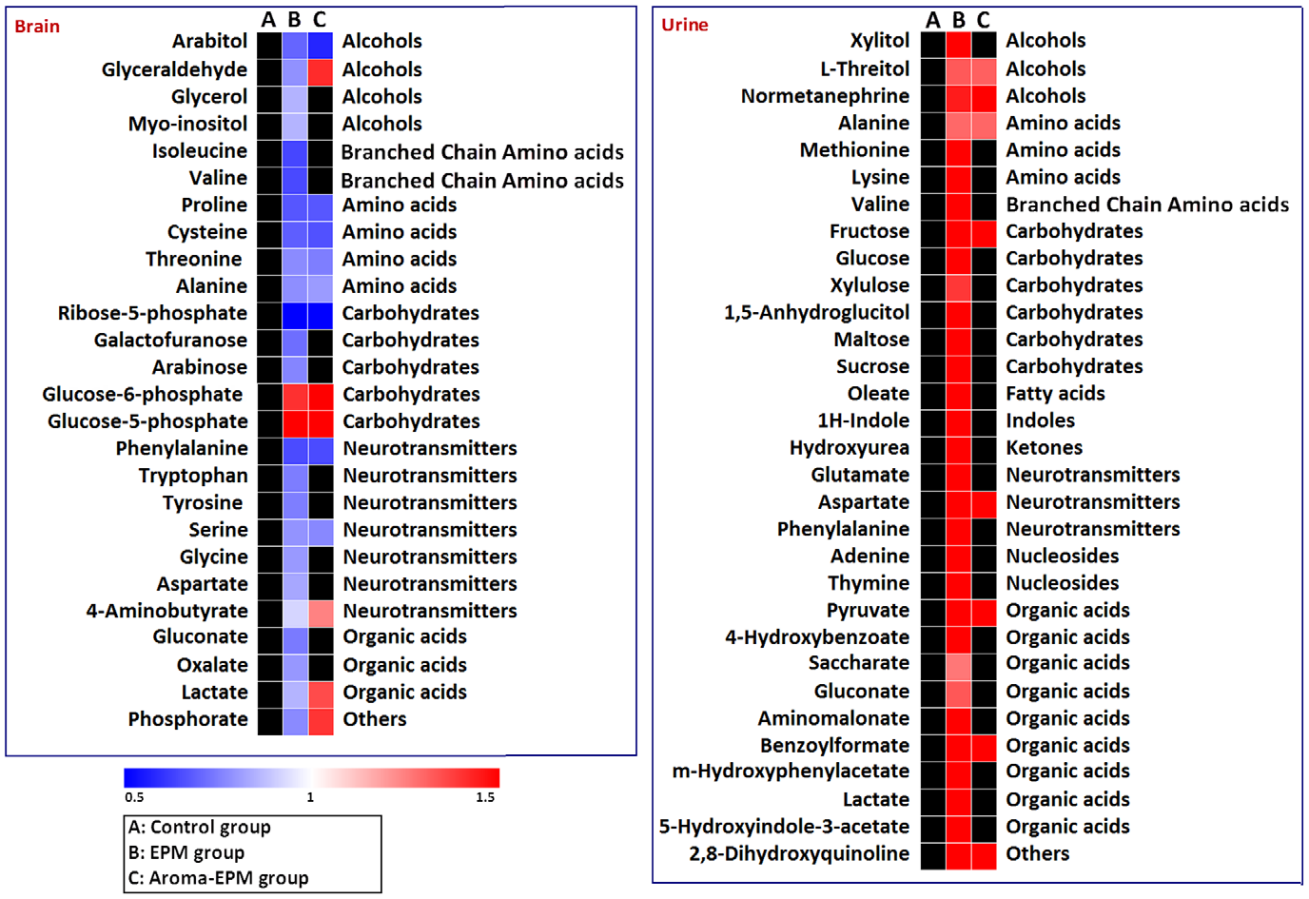
Heatmaps show changes in metabolites compared to controls at EPM group, aroma-EPM group. Shades of red and blue represent fold increase and fold decrease of a metabolite, respectively, in EPM group or aroma-EPM group relative to control group (see color scale).

Statistical analysis of brain tissue and urinary profiles revealed significant metabolic changes associated with EPM test (**Table 2**, **Figure 4**). As compared to controls, those rats that underwent EPM test were characterized with lower levels of neurotransmitters (GABA, phenylalanine, tryptophan, tyrosine, serine, glycine, aspartate), carbohydrates (galactofuranose, ribose-5-phosphate and arabinose), amino acids (isoleucine, valine and proline), and organic acids (oxalate and lactate) in brain tissues (**Table 2**, **Figure 4A**), but showed a higher urinary concentration of neurotransmitters (aspartate, glutamate and phenylalanine), carbohydrates (fructose, glucose and xylulose), amino acids (valine, alanine, etc.), organic acids (pyruvate, etc.), fatty acids (oleate) and gut microbial activity (4-hydroxybenzoate, m-hydroxyphenylacetate, and 5-hydroxyindole-3-acetate) (**Table 2**, **Figure 4B**).

### Anxiolytic Effects of Aromas on EPM induced Anxiety Model Rats

The EPM induced metabolic differences observed in urine or brain tissue was significantly reduced after 10 days of aroma inhalation, as noted with the loss of statistical significance on many of the metabolites in the aroma-EPM group (**Figure 4** and **Table 2**). The heatmaps in **Figure 4** showed that significantly altered metabolites in EPM group were attenuated in aroma-EPM group (non-significant fold changes are in black).

## Discussion

In the present study, MS-based metabolite profiling provided a direct visualization of the global metabolic responses of rats to a daily inhalation of essential oil. The therapeutic use of essential oils is an inexpensive and noninvasive complementary medicine practice that dates back centuries to improve wellbeing [19]. The proposed mechanism of action of the respiratory administration of aromas begins with the absorption of volatile odor molecules through the nasal mucosa [20]. Odor molecules are then transformed into chemical signals, which travel to the olfactory bulb and then other parts of the limbic system of the brain and the cerebral cortex and the olfactory sensory center at the base of the brain, interacting with the neuropsychological framework to produce characteristic physiological and psychological effects on target tissues [20]. Through this whole mechanism the brain comes into direct contact with the outside world. In addition, the most prevalent mental disorder, anxiety disorder, is associated with both functional and morphological brain changes that commonly involve the ‘fear network’ including the prefrontal cortex, hippocampus and amygdala [21], [22]. Hence the brain tissue, as a whole, together with biofluids such as urine, produces a subtle but identifiable metabonomic change in response to aroma inhalation within a time course, which is detected by a metabonomic approach. In addition, we have not detected essential oil ingredients in rat brain tissues and urine samples, presumably due to the fact that those volatile organic compounds are of too low concentration through aerial diffusion and subsequent inhalation to have a detectable level in biofluids and tissues in rats.

### Inhalation of Aromas is Associated with Specific Brain and Urine Metabolic Signatures

Because of the blood-brain barrier, the metabolism of the brain is independent of peripheral circulation, and as a result, the metabolic variances induced by aromas inhalation were different between urine and brain (**Table 1**). The high urine levels of metabolites may indicate excessive loss of the metabolites in the brain. An interesting finding of our analysis suggests the increased carbohydrates in the brain that are critically involved in the metabolic perturbation derived from inhalation of aromas. Carbohydrates have been shown in several reports that have anxiolytic effects [23] and the significantly increased levels of carbohydrates may be due to the therapeutic effects of essential oils on anxiety and depression. It is believed that the decreased levels of brain histamine, which are associated with a functional polymorphism of histamine N-methyltransferase (HNMT) called Thr105 allele, may result in higher levels of anxiety [24]. Phenylalanine can be biosynthesized to phenylethylamine (PEA) by enzymatic decarboxylation. High levels of PEA are observed in individuals experiencing “mind racing”, such as sleep problems, anxiety, and schizophrenia [25]. Our findings of the increased histamine and decreased phenylalanine levels in brain after aromas inhalation further support the therapeutic effects of essential oils on anxiety. A great many neurotransmitters in rat brain were significantly decreased after inhalation of essential oils, although only aspartate was significantly increased in rat urine.

It was interesting to find that fatty acids, including methyl arachidonate, linolate, docosahexaenoate, oleate, palmitate, stearate, palmitoleate and arachidonate, were all down-regulated in brain after essential oil inhalation (**Table 1**). It was reported that carbohydrate metabolism will promote fatty acid oxidation [26], and fatty acids can also be synthesized from carbohydrates. Carbohydrates have first been catabolized into acetyl CoA, which will be used to synthesize fatty acids in a process known as lipogenesis [27]. The decreased fatty acids in rat brain tissue may be due to the significantly increased carbohydrates after inhalation of essential oils.

### The Biological Response of Rats Undergoing EPM was Dependent on Aromas Induced Metabolic Variations

The EPM is a widely used behavioral test for rodents to assess the anti-anxiety effects of pharmacological agents and to define brain regions and mechanisms underlying anxiety-related behavior. As compared to the control group, the two significantly increased primary anxiety-like response indices (OE% and OT%) (**Figure 1**) in the aroma group of EPM test (p<0.05) indicates the anxiolytic activity by the inhalation procedure, which is consistent with other published studies [28], [29].

Differentially expressed metabolites associated with EPM were characterized by GC-TOFMS based metabonomics approach, including amino acid neurotransmitters (glycine, GABA, aspartate, *etc.*), carbohydrates, alcohols, nucleosides, organic acids and amino acids (valine and isoleucine). All these metabolites in brain tissues and urine were grouped into several types, with their variation induced by EPM and the aromas intervention, as seen in **Figure 4**.

Anxiety disorder is mainly associated with both functional and morphological brain changes, and the altered level of neurotransmitters can be readily seen in brain tissues. In brain tissues of EPM rats, neurotransmitters, aspartate, glycine, tryptophan, serine, phenylalanine, tyrosine, and GABA were significantly decreased, while in urine, the three neurotransmitters, glutamate, aspartate and phenylalanine were significantly increased. It is believed that low levels of these neurotransmitters in the brain may result in anxiety-related disorders and high urine levels of neurotransmitters may indicate excessive loss of the neurotransmitters in the brain. As for carbohydrates, the levels of D-ribose-5-phosphate, arabinose, and D-galactofuranose were significantly decreased while the levels of glucose-5-phosphate and glucose-6-phosphate were significantly increased in brain. An altered carbohydrate metabolism revealed by an upregulation of glycolysis and the TCA cycle was shown to be significantly related to the anxiety-like behavioral phenotype [30]. EPM model rats have lower levels of myo-inositol than controls. Myo-inositol has been shown to have anxiolytic activities in both humans and animals [31], [32]. Also of interest is the finding that several energy metabolism related metabolites, including amino acids, oxalate, and lactate, were found at different levels between the EPM group rats and controls. Oxidative stress has been found to be involved in the pathogenesis of neurological diseases, such as psychiatric disorders [33], [34]. Human studies on panic disorder and obsessive-compulsive disorder also suggest an involvement of oxidative stress in anxiety [35], [36]. Oxidative stress is caused by altered mitochondrial energy pathways leading to abundant reactive oxidative stress compounds. It is therefore not surprising that metabolites and carbohydrates involved in TCA cycle were found to be significantly different between the EPM group rats and controls. It was also reported that stress-induced anxiety in mice leads to elevated levels for glucose 6-phosphate in the brain [37] that can result in excessive oxidative damage. Reciprocal changes in glucose 6-phosphate levels in the brain reflect the rate of glycolysis in the Embden-Myerh of pathway at the site of action of phosphofructokinase [38].

It is believed that there was a strong relationship between gastrointestinal symptoms, anxiety disorders and depression [39]. Changes of gastrointestinal functional ecology are intimately linked to gut microbial populations and activities [40]. Urine of mammals contains many polar metabolites resulting from gut microbial-mammalian co-metabolism [41], [42]. Therefore, metabolic variations of urinary excretion of many aromatic compounds (e.g., phenolics, indoles and benzoyl derivatives) provide indirect information on the gut microbial metabolic activities [42]. Urinary 4-hydroxybenzoate, m-hydroxyphenylacetate, 1H-indole, and 5-hydroxyindole-3-acetate were all significantly increased in EPM group rats, reflecting an altered gut microbial metabolism associated with aroma inhalation. Certain aromatic compounds, such as benzoate and phenylacetate, can be regarded as scavenging agents for endogenous glycine and glutamine [43], [44]. Metabolite profiling of urine in this study revealed relatively lower levels of glycine and glutamine and higher levels of hippurate without significance (data not shown) in EPM group rats.

### Anxiolytic Effects of Aromas on Rats Subjected to EPM Test

The metabolic profiling reveals the global metabolic perturbation associated with EPM-induced anxiety and aromas intervention. The heatmaps (**Figure 4**) generated with the differential metabolites contributing to the separation of EPM group and aroma-EPM group from the control group indicate less significant fluctuation of metabolite levels in the aroma-EPM group. This suggests that aromas inhalation attenuated the EPM-induced metabolic perturbation in rats. The inhibition of metabolic alteration by aromas is consistent with EPM behavioral test as demonstrated by the significantly increased OT% and OE% in aroma-EPM group.

The essential oil made from *Lavandula angustifolia* [45], *Salvia sclarea L., Santalum album,* and *Citrus sinensis* has been reported to be both efficacious and safe for the relief of anxiety disorder [2]. Essential oils, given systemically or focally into the dorsal hippocampus of a rat, affect the synaptic concentrations of some excitatory and inhibitory amino acid neurotransmitters [46]. The mechanisms underlying the anxiolytic activities of essential oils may be due to alterations in GABA function, the major inhibitory neurotransmitter in the brain. The increased GABA levels in the brain are supported by a previous report showing that inhalation of linalool, the lavender essential oil odor, can potentiate the action of GABA by inhibiting the opposing excitatory neurotransmitter system [47]. It has been suggested that anxiety disorders are associated with dysfunction of GABAergic systems possibly due to reduced receptor sensitivity and/or decreased levels of the neurotransmitter [48]. Another study showed that lavender oil, mainly containing linalyl acetate and linalool, potentiated the GABAA receptor response caused by a low concentration of GABA, suggesting the anxiolytic, anticonvulsant and sedative activity [49]. These two compounds are also the main components in the essential oil used in this study. Therefore, further studies are needed to investigate whether the anxiolytic properties of this essential oil are mediated by GABAA receptors.

The aim of this study was to obtain metabolite markers in brain tissue and urine of experimental rats exposed to essential oil to gain mechanistic insights into the metabolic impact of aromas exposure. However, there are limitations in the current GC-TOFMS based metabonomic study. First, only GC-TOFMS based metabonomics platform was used to characterize the metabolic responses to aromas intervention in brain tissue and urine. Additional mechanistic information and metabolite markers may be identified with an improved analytical and metabolite identification capability in the future study. Secondly, the underlying mechanisms of the observed changes in metabolic phenotype warrant further investigation with a goal to verify potential candidate biomarkers in rats/human afflicted with anxiety disorders and test new medications for their treatment.

In conclusion, we identified the global metabolic responses to aromas intervention characterized by unique metabolic signatures in rat brain tissue and urine involving neurotransmitters, fatty acids, carbohydrates and amino acids. Inhalation of essential oil is able to attenuate anxiety-induced metabolic perturbation, concurrent with the behavioral findings that inhalation of essential oil significantly increased the open arms time and open arms entries.

## Supporting Information

Table S1 Chemical composition of the essential oil.
(DOCX)
