# Supplementary material for: The Metabolic Responses to Aerial Diffusion of Essential Oils
Source: PLoS One. 2012 Sep 12;7(9):e44830. doi: 10.1371/journal.pone.0044830 (PMC3440318; doi:10.1371/journal.pone.0044830)
Supplement: Table S1 — Chemical composition of the essential oil. (DOCX) [file pone.0044830.s001.docx]

**Supporting Information**

**Table S1.** Chemical composition of the essential oil.

| **No.** | **Component** | ***I^T^*** | **RRI** | **Relative content (%)** |
| --- | --- | --- | --- | --- |
| 1 | Tricyclene | 1017 | 1014 | 0.23 |
| 2 | α-Pinene | 1035 | 1027 | 0.02 |
| 3 | Myrcene | 1163 | 1174 | 0.68 |
| 4 | Limonene | 1204 | 1203 | 24.51 |
| 5 | Isoamyl alcohol | 1207 | 1212 | 0.19 |
| 6 | 1,8-Cineole | 1209 | 1213 | 0.41 |
| 7 | (E)-2-Hexenal | 1239 | 1232 | 0.76 |
| 8 | γ-Terpinene | 1256 | 1255 | 0.42 |
| 9 | p-Cymene | 1277 | 1280 | 0.2 |
| 10 | α-Pinene oxide | 1384 | 1384 | 0.32 |
| 11 | Chrysanthenone | 1526 | 1522 | 0.37 |
| 12 | Linalool | 1556 | 1553 | 16.42 |
| 13 | Linalyl acetate | 1569 | 1565 | 24.7 |
| 14 | β-Elemene | 1604 | 1600 | 1.63 |
| 15 | β-Caryophyllene | 1608 | 1607 | 0.97 |
| 16 | Terpinen-4-ol | 1612 | 1611 | 0.32 |
| 17 | trans-p-Mentha-2,8-dien-1-ol | 1678 | 1678 | 0.31 |
| 18 | α-Terpineol | 1703 | 1706 | 1.59 |
| 19 | α-Terpinyl acetate | 1708 | 1709 | 0.3 |
| 20 | Borneol | 1716 | 1719 | 0.18 |
| 21 | Neryl acetate | 1732 | 1733 | 0.4 |
| 22 | Geranyl acetate | 1763 | 1765 | 0.74 |
| 23 | γ-Cadinene | 1779 | 1776 | 0.12 |
| 24 | Nerol | 1806 | 1808 | 0.31 |
| 25 | cis-Calamenene | 1853 | 1853 | 0.82 |
| 26 | Polysantol | 1919 | / | 11.84 |
| 27 | Isocaryophyllene oxide | 2000 | 2001 | 0.34 |
| 28 | Caryophyllene oxide | 2013 | 2008 | 0.11 |
| 29 | Germacrene D-4-ol | 2071 | 2069 | 0.12 |
| 30 | 1-*epi*-Cubenol | 2088 | 2088 | 0.84 |
| 31 | Elemol | 2097 | 2096 | 0.15 |
| 32 | Spathulenol | 2139 | 2144 | 0.13 |
| 33 | (Z)-3-Hexenylbenzoate | 2148 | 2148 | 0.13 |
| 34 | β-Eudesmol | 2164 | 2257 | 2.23 |
| Total |  |  |  | 92.81 |

RRI：Retention indices on an innowax column
